# Effect of Liuweiwuling tablet on biochemical and virological parameters, and quality of life in patients with hepatitis B virus-related cirrhosis: A protocol for a systematic review and meta-analysis

**DOI:** 10.1097/MD.0000000000022065

**Published:** 2020-09-11

**Authors:** Ding Li, Min Zhu, Changhui Zhou, Xiujing Liu

**Affiliations:** a Department of Clinical Laboratory; b Department of Central Laboratory, Liaocheng People's Hospital, Liaocheng, Shandong Province, P.R. China.

**Keywords:** efficacy, hepatitis B virus-related cirrhosis, Liuweiwuling tablet, meta-analysis

## Abstract

**Background:**

Liuweiwuling (LWWL) tablet, a kind of plant-derived traditional Chinese medicine preparation, has been widely applied as a promising adjunctive drug for hepatitis B virus-related cirrhosis (HBVC). However, its exact clinical efficacy and safety is still not well investigated. In this study, we aimed to summarize the efficacy of LWWL tablet on biochemical and virological parameters, and quality of life (QoL) in patients with HBVC through the meta-analysis.

**Methods:**

All available randomized controlled trials and high-quality prospective cohort studies that investigated the efficacy and safety of LWWL for patients with HBVC were searched from the following electronic databases: PubMed, Medline, Cochrane Library, Google Scholar, Web of Science, Excerpt Medica Database, China National Knowledge Infrastructure, Chinese Biomedical Literature Database, China Scientific Journal Database, and Wanfang Database. Papers in Chinese or English published from January 2000 to August 2020 will be included without any restrictions.

Study selection and data extraction will be performed independently by 2 authors. The clinical outcomes including biochemical (liver function and fibrosis indexes) and virological parameters, QoL, immune function and adverse events, were systematically evaluated. Review Manager 5.3 and Stata 14.0 were used for data synthesis, sensitivity analysis, meta regression, subgroup analysis, and risk of bias assessment.

**Results:**

The results of this study will be published in a peer-reviewed journal, and provide a helpful evidence for clinicians to formulate the best postoperative adjuvant treatment strategy for HBVC patients.

**Conclusion:**

Our study will draw an objective conclusion of the efficacy of LWWL on biochemical and virological parameters, and QoL in patients with HBVC.

**INPLASY registration number:**

INPLASY202080010.

## 1 Introduction

### 1.1 Description of the background

Liver cirrhosis (LC, being the 14th most common cause of death worldwide) is an increasing cause of morbidity and mortality in more developed countries, which usually results from prolonged or repeated alcohol excess, viral hepatitis, and other etiologies.^[1–5]^ There are over 350 million people with chronic hepatitis B worldwide, and hepatitis B virus (HBV) infection is one of the main causes of LC and hepatocellular carcinoma (HCC).^[6,7]^ More than one million HBV carriers die of LC and HCC every year.^[7]^ As reported by the World Health Organization, 45% of the population lives in high-prevalence chronic hepatitis B areas.^[7,8]^ Among untreated chronic hepatitis B patients, 8% to 20% develop to cirrhosis in 5 years.^[7,9,10]^ Meanwhile, untreated decompensated cirrhosis patients show a poor prognosis, with a 5-year survival rate ranging from 14% to 35%.^[7,9,10]^

### 1.2 Description of the intervention

Currently, clinical treatments for hepatitis B virus-related cirrhosis (HBVC) are orally administered nucleotide analogs (NAs), such as entecavir, adefovir dipivoxil, tenofovir, lamivudine, and telbivudine.^[11–15]^ As NAs suppress HBV replication only at the point of deoxyribonucleic acid (DNA) synthesis progression, most patients require long-term treatment.^[7,16]^ However, prolonged use of NAs may lead to drug resistance and kidney impair, the most importantly, eradication or suppression of HBV does not reverse the fibrosis of liver and eliminate the risk of occurrence of HBVC.^[11,12]^ Therefore, exploring new alternative regimens for patients with HBVC are urgently required.

Traditional Chinese Medicine has been clinically used as a complementary treatment for various diseases including HBVC, and plays an important role in the health of Asian people.^[17–19]^ Many scholars pointed out that the combination of Chinese and Western medicine may be the potential trend for refractory disease treatment in the future.^[17–19]^ Liuweiwuling (LWWL) tablet is a famous plant-derived traditional Chinese medicine, which composed of 6 traditional Chinese herbs: *Schisandrae chinensis fructus, Fructus Ligustri Lucidi, Forsythiae fructus, Curcumae rhizoma, Perennial sow thistle, and Ganoderma lucidum spore* in a ratio of 3.5:2.5:1.5:1:1.5:1, respectively.^[20–24]^ Modern pharmacological studies have shown that *Schisandra chinensis* is effective in reducing enzymes and in protecting the liver from chronic liver injury^[21,22]^; *Ligustri lucidi fructus* nourishes the liver and kidneys^[21,22]^; *Forsythiae fructus* and *Perennial sow thistle* have a positive effect on clearing heat and detoxifying for the liver^[21,22]^; *Curcumae rhizoma* can desilt the extravasated blood and promote microcirculation^[21,22]^; and *Ganoderma lucidum spore* can enhance immunity and promote tissue repair.^[21,22]^ Lei et al^[25]^ pointed out that LWWL tablet can protect hepatocytes from liver injury by decreasing the levels of alanine aminotransferase (ALT)/aspartate aminotransferase (AST) and the release of inflammatory cytokines, such as high mobility group box protein B1, tumor necrosis factor-α, and interleukin-1β, and promoting liver regeneration.

Several preclinical studies have demonstrated that LWWL tablet are effective against a variety of acute or chronic liver injuries that are caused by drugs, viruses, and alcohol.^[20–24]^ It is effective in preventing the progression of hepatic fibrosis and cirrhosis, and when combined with antivirals, may produce improved clinical effects.^[17,26,27]^ Despite the intensive clinical studies, its clinical efficacy was still not well investigated. In this study, we are prepared to summarize the efficacy of LWWL on biochemical and virological parameters, and quality of life (QoL) in patients with HBVC through the meta-analysis, in order to provide a helpful evidence for clinicians to formulate the best postoperative adjuvant treatment strategy for HBVC patients.

## 2 Review question

Is LWWL tablet effective on biochemical and virological parameters, and QoL in patients with HBVC?

## 3 Objective

A systematic review and meta-analysis will be performed to systematically evaluate the efficacy of LWWL tablet on biochemical and virological parameters, and QoL in patients with HBVC.

## 4 Methods

The protocol of this meta-analysis will be reported according to Preferred Reporting Items for Systematic Review and Meta-Analysis Protocols guidelines.^[28]^ Our protocol has been registered on the International Platform of Registered Systematic Review and Meta-Analysis Protocols (INPLASY). The registration number was INPLASY202080010 (DOI number is 10.37766/inplasy2020.8.0010, https://inplasy.com/inplasy-2020-8-0010/). This meta-analysis is a secondary research which based on some previously published data. Therefore, the ethical approval or informed consent was not required.

### 4.1 Search strategy

To perform a comprehensive and focused search, experienced systematic review researchers will be invited to develop a search strategy. The plan searched terms are as follows: “hepatitis B virus” or “liver cirrhosis” or “hepatitis B cirrhosis” or “hepatitis B virus-related cirrhosis” or “HBV-cirrhosis” or “HBV-C” combined with “Liuweiwuling” or “Liuweiwuling tablet” or “LWWL” et al. An example of search strategy for PubMed database shown in Table 1 will be modified and used for the other databases.

**Table 1:**
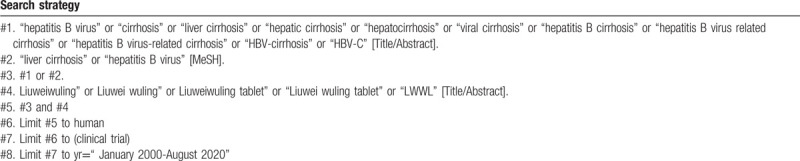
Searching strategy in PubMed.

### 4.2 Eligibility criteria

#### 4.2.1 Types of studies

All available randomized controlled trials (RCTs) or quasi-RCTs, and high-quality prospective cohort studies that investigated the efficacy of LWWL tablet on biochemical and virological parameters, and QoL in patients with HBVC will be included in this systematic review.

#### 4.2.2 Types of participants

Patients were diagnosed hepatic cirrhosis that caused by HBV. No restrictions regarding age, gender, racial, region, education, and economic status in this analysis. Patients who had no HCC or other malignant tumor

#### 4.2.3 Types of interventions

HBVC patients in the experimental group must be treated with conventional treatment (including routine liver protection therapy or oral antiviral therapy,) combined with LWWL tablet.

#### 4.2.4 Comparator

In the control group, HBVC patient treated with the same conventional treatment as experimental group.

#### 4.2.5 Exclusion criteria

Papers without sufficient available data, non-peer reviewed studies, noncomparative clinical trials, literature reviews, meta-analysis, meeting abstracts, case reports, commentaries, letter to the editor, and other unrelated researches will be excluded from analysis.

### 4.3 Information sources

Electronic databases including PubMed, Medline, Cochrane Library, Google Scholar, Web of Science, Excerpt Medica Database, China National Knowledge Infrastructure, Chinese Biomedical Literature Database, China Scientific Journal Database, and Wanfang Database will be systematically searched for eligible clinical trials from January 2000 to August 2020. Language is limited with English and Chinese.

### 4.4 Types of outcome measures

#### 4.4.1 Primary outcomes

- Liver function indexes: Liver function of patients with HBVC was assessed in terms of total bilirubin, serum albumin, ALT, and AST levels;
- Virological indicators: HBV DNA levels, Hepatitis B virus e antigen (HBeAg) status, and seroconversion;
- QoL as evaluated by Karnofsky score;

#### 4.4.2 Secondary outcomes

Secondary outcomes will include:

- Model for end-stage liver disease and Child-Pugh score.
- Alpha-fetoprotein level;
- Liver fibrosis indexes: hyaluronic acid, laminin, type III procollagen, and type IV collagen levels.
- Immune function indicators: CD3^+^, CD4^+^, CD8^+^, NK cells percentage, and CD4+/CD8+ cell ratios;
- Adverse events: toxicity was graded from 0 to IV in severity on the basis of the World Health Organization recommendations.

### 4.5 Data collection and analysis

We will adopt the measures described in the Cochrane Handbook for Systematic Reviews of Interventions to pool the evidence.^[29]^

#### 4.5.1 Study selection and management

**Figure 1:**
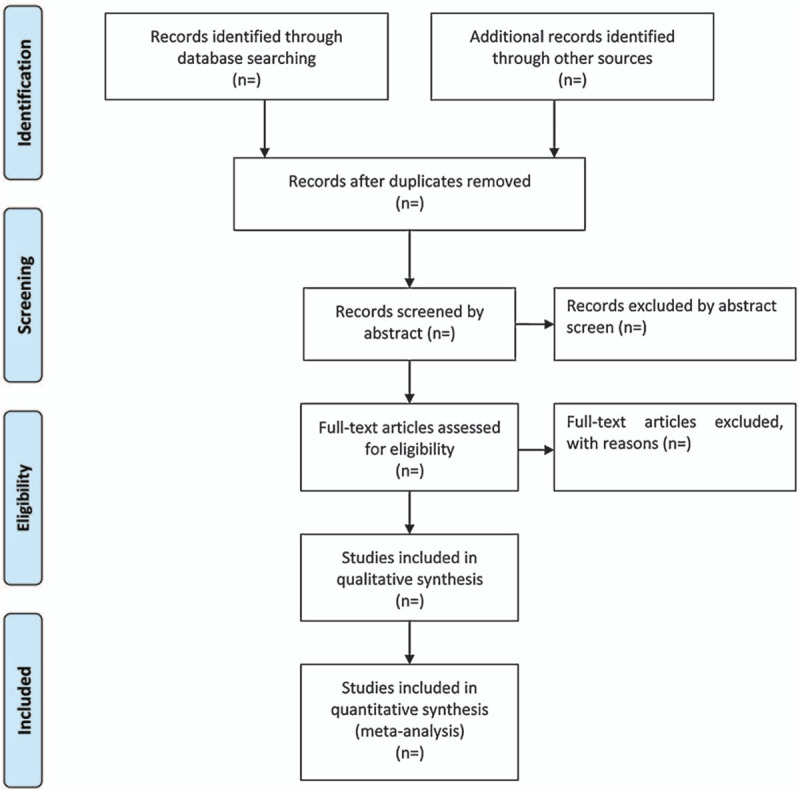
Study selection process for the meta-analysis.

Two authors (Li D and Zhu M) will be reviewed independently to identify potential trials by assessing the titles and abstracts and identify whether the trials meet the inclusion criteria. The full text will be further reviewed to exclude irrelevant studies or determine potential eligible studies. Endnote X7 software will be used for literature managing and records searching. Disagreements between the 2 reviewers will be resolved by discussing with the third investigator (Zhou CH). Excluded studies and the reasons for exclusion will be listed in a table. A Preferred Reporting Items for Systematic Review and Meta-Analysis-compliant flow chart (Fig. 1) will be used to describe the selection process of eligible literatures.

#### 4.5.2 Data extraction and management

Two investigators (Li D and Zhu M) will be responsible for the data extraction independently according to the Cochrane Handbook for Systematic Reviews of Intervention.

The following data will be extracted from eligible literatures:

- Study characteristics and methodology: country of study, the first author, year of publication, study design, sample size, periods of data collection, total duration of study, follow-up duration, and so on.
- Participant characteristics: age, gender, ethnicity, inclusion and exclusion criteria, and so on.
- Interventions: therapeutic means, manufacturer of the drugs, dosage of LWWL tablet, administration route and cycles, duration of treatment, follow-up time, and so on.
- Outcome and other data:
  (1) Liver function indexes (total bilirubin, albumin, ALT, and AST);
  (2) Virological indicators (HBV DNA levels and HBeAg status);
  (3) Liver fibrosis indexes (hyaluronic acid, laminin, type III procollagen and type IV collagen levels);
  (4) Immune indicators: (CD3^+^, CD4^+^, CD8^+^, NK cells percentage, and CD4+/CD8+ cell ratios);
  (5) Model for end-stage liver disease and Child-Pugh score;
  (6) Other data (QoL, alpha-fetoprotein, adverse effects, etc).

Dealing with missing data: we will attempt to contact the authors to request the missing or incomplete data. If those relevant data are not acquired, they will be excluded from the analysis.

### 4.6 Assessment of risk of bias

Two review authors (Li D and Zhu M) will independently assess the methodological quality of the included trials by using the following criteria described in the Cochrane Handbook for Systematic Reviews of Interventions: random sequence generation, allocation concealment, blinding of participants and personnel, blinding of outcome assessment, incomplete outcome data, selective reporting, and other bias.^[29,30]^ Evidence quality will be classified as low risk, high risk, or unclear risk of bias. Effective Practice and Organisation of Care guidelines will be used to assess the risks of non-RCTs.^[31]^ Any disagreements will be resolved via discussion with a third researcher (Zhou CH).

### 4.7 Data synthesis

Review Manager 5.3 (Nordic Cochran Centre, Copenhagen, Denmark) and Stata 14.0 (Stata Corp., College Station, TX) statistical software will be used to pool the data and carry out the data analysis. Heterogeneity between studies will be assessed using the Cochran *Q* and Higgins *I*^*2*^ statistic. *P* < .1 or *I*^*2*^ > 50% will be considered as showing considerable heterogeneity, and the random effect model will be used.^[32]^ Otherwise, a fixed effect model will be used for data analysis. Dichotomous data will be recorded as risk ratio with 95% confidence intervals. Continuous data will be presented as standardized mean difference with their confidence intervals. A 2-tailed *P* < .05 was considered statistically significant.

### 4.8 Subgroup and meta-regression analysis

Subgroup and meta-regression analysis based on cirrhosis stage, treatment period, dose of LWWL tablet, level of risk of bias, and other unpredictable factors will be performed to detect the source of heterogeneity.

### 4.9 Sensitivity analysis

Sensitivity analysis will be carried out to assess the reliability and robustness of the pooled results via eliminating trials with low quality. A summary table will report the results of the sensitivity analyses.

### 4.10 Other relevant information

#### 4.10.1 Publication bias analysis

Funnel plot, Begg, and Egger regression test will be performed to analyze the existence of publication bias if 10 or more studies are included in this meta-analysis.^[33–35]^ If reporting bias is suspected, we will consult the study author to get more information. If publication bias existed, a trim-and-fill method should be applied to coordinate the estimates from unpublished studies, and the adjusted results were compared with the original pooled risk ratio.^[36]^

#### 4.10.2 Evidence evaluation

The Grading of Recommendations, Assessment, Development, and Evaluation system will be used for assessing the strength of the body of evidence.^[37]^ According to the grading system, the quality of evidence will be evaluated as high, moderate, low, and very low level.

### 4.11 Dissemination plans

We will disseminate the results of this systematic review by publishing the manuscript in a peer-reviewed journal.

## 5 Discussion

HBV infection is one of the most common chronic viral infections in the world, which causes 40% of men and 15% of women to die of LC or HCC.^[12,38]^ Timely and effective treatment of HBVC at the compensatory stage could prevent cirrhosis transformation to decompensated cirrhosis and HCC.^[11]^ At present, the recognized therapeutic strategies are antiviral therapy for etiology and direct antifibrotic therapy for extracellular matrix, metabolism, and hepatic stellate cell activation.^[11,39]^ However, antiviral therapy alone cannot inhibit the formation and deposition of extracellular matrix in liver fibrosis tissues, and single anti-fibrosis therapy cannot inhibit the replication of hepatitis virus and promote the repair of liver injury.^[11]^ Evidence-based medicine has shown that antiviral therapy plus traditional Chinese medicine has better long-term control effect on HBVC than antiviral treatment alone.^[11,12,40,41]^ LWWL tablet is a famous plant-derived traditional Chinese medicine preparation against hepatic fibrosis and cirrhosis which produced by Shandong Shibo Jindu Pharmaceutical Co., Ltd. It have been approved by Chinese State Food and Drug Administration, and granted the manufacturing approve number accordingly (Z20060238).

## 6 Strengths and limitations

Several studies have shown that LWWL tablet has a reliable effect on improving the pathological state of cirrhosis and delaying the formation of liver fibrosis.^[26,27]^ Even though there was statistical analysis of published clinical trials, the exact effects of LWWL tablet on biochemical and virological parameters, and QoL in patients with HBVC were still not systematically investigated. This systematic review will conduct a systematic, comprehensive, and objective evaluation of LWWL-based treatment. The findings of this analysis will provide a helpful evidence for clinicians to formulate the best postoperative adjuvant treatment strategy for patients with advanced HBVC, and also provide scientific clues for researchers in this field. There may be a language bias with the limitation of English and Chinese studies.

## Author contributions

**Conceptualization:** Xiujing Liu and Ding Li.

**Data curation:** Ding Li and Min Zhu.

**Formal analysis:** Ding Li and Min Zhu.

**Funding acquisition:** Changhui Zhou.

**Investigation:** Ding Li, Min Zhu and Changhui Zhou.

**Methodology:** Ding Li, Min Zhu and Changhui Zhou.

**Project administration:** Xiujing Liu.

**Resources:** Xiujing Liu and Ding Li.

**Software:** Xiujing Liu and Ding Li.

**Supervision:** Xiujing Liu and Ding Li.

**Validation:** Xiujing Liu and Changhui Zhou.

**Visualization:** Ding Li and Min Zhu.

**Writing – original draft:** Ding Li, Min Zhu and Changhui Zhou.

**Writing – review & editing:** Xiujing Liu and Changhui Zhou.
